# Intention to consume news *via* personal social media network and political trust among young people: The evidence from Hong Kong

**DOI:** 10.3389/fpsyg.2022.1065059

**Published:** 2023-01-12

**Authors:** Youliang Zhang, Zhen Tian, Ziwei Zhou, Jing Huang, Alex Yue Feng Zhu

**Affiliations:** ^1^Institute of Higher Education, Beijing University of Technology, Beijing, China; ^2^School of Graduate Studies, Lingnan University, Hong Kong SAR, China; ^3^Institute of Policy Studies, Lingnan University, Hong Kong SAR, China

**Keywords:** news, political trust, social media, young people, Hong Kong

## Abstract

**Introduction:**

Assessing the effect of different media sources on political trust provides an overall picture of the role of the current media landscape in influencing the legitimacy of political institutions.

**Methods:**

A cross-lagged model was developed and tested by applying it to a three-wave dataset obtained by surveying young people in Hong Kong in order to evaluate the unique impact of intention to consume news *via* personal social media network on political trust.

**Results:**

After controlling for the impact of other news channels and respondents’ prior political interest, we found their political trust was most significantly affected by information intentionally obtained from friends and family through their personal social media network.

**Discussion:**

Media exhibits a relatively weak effect on political trust, which is primarily influenced by selection, indicating that policymakers do not have to be concerned with online exchange of information that is critical of the government. Moreover, this evidence from Hong Kong suggests that, when it comes to developing political trust throughout the society, conflicts resulting from opposing views are better than ignorance.

## 1. Introduction

Political trust, also known as trust in government, reflects citizens’ reliance on or confidence in their political leadership, institutions, and legitimacy (Newton, 2001; Fan, 2019). Thus, it is particularly important in times of crisis and societal change (Armingeon and Guthmann, 2014; Arpino and Obydenkova, 2020; Zhang et al., 2022), as it enhances public compliance with the laws and reduces the risk of dissention (Levi and Stoker, 2000; Lalot et al., 2022) while facilitating implementation of public policies (Francis, 2016; Xiong, 2016). Given the critical role of political trust in sustaining democracy, it is typically associated with its legitimacy (Easton, 1975). In other words, political legitimacy of a regime is likely to be questioned if citizens demonstrate low political trust and show little interest in engaging in civic activities.

Political trust is a critical issue in Hong Kong due to the considerable changes to its political and socioeconomic systems throughout its history (Cheung, 2013). Since the issuance of the Sino−British Declaration in 1997, Hong Kong city has been subject to “one country, two systems” policy in order to maintain the success of the economy and society (Zhang and Ip, 2019). A recent study, however, revealed that political trust among Hong Kong citizens experienced considerable fluctuations between 1992 and 2019, with a progressive reduction after 2007 (University of Hong Kong, 2020). As citizens’ political trust is influenced by the government’s capacity and effectiveness in providing public services, it is widely believed that its decline in recent decades in Hong Kong is due to the growing urban poverty (Kwong, 2016). This view is also supported by the growing number of social movements and events that have taken place in Hong Kong in recent years, in which those aged 15–24 years predominated (Ma, 2015; Chan, 2016; Kwong, 2016).

News consumption has long been recognized as a key determinant of political trust (Hanitzsch and Berganza, 2012; Tworzecki and Semetko, 2012). News consumptions *via* both “traditional” and “emerging social media” are consistently documented to cause opinion polarization and reducing political trust. Information provided by traditional media can be selectively consumed if it is inconsistent with individual attitudes, emotions and beliefs (Arceneaux and Johnson, 2013; Weeks et al., 2015; Mummolo, 2016; Anspach, 2017), giving rise to biased information processing (Taber and Lodge, 2006; Lodge and Taber, 2013). As Zhu et al. (2020) indicated, new consumption in social media led to the construction of different user groups, each of which had its own like-minded political orientation. Hence, news consumption *via* social media created boundaries among users, which is linked to more radical political attitudes (Barberá, 2015; Bail et al., 2018; Lee et al., 2018; Wang, 2018; Zhu et al., 2020).

Previous research, however, was mostly conducted to examine “new consumption” in social media as the predictor of political trust among young people. This study aims to investigate “the intention to consume news” *via* personal social media network, which is a relatively underexplored factor impacting their political trust. We gathered pertinent data by conducting a three-wave survey in Hong Kong, as this is a particularly relevant context for the exploration of this phenomenon. In our model, we controlled for the impact of intention to consume news through other means as well as political interest, to eliminate the influence of these factors on our findings (Ceron, 2015; Strömbäck et al., 2016; Meng and Zhou, 2022).

## 2. Background

As Hong Kong is a special administrative region of China, political trust of its citizens is of particular importance, as it is believed to reflect their confidence in the “one country, two systems” institution (Zhang and Ip, 2019). Since 2014, a growing number of citizens have shown their distrust in the Hong Kong’s political institutions and have started to engage in social activities such as the Umbrella Movement and Water Movement. Moreover, a significant proportion of those involved have been young people aged 15–24 years (Chan, 2016; Kwong, 2016; Chinese University of Hong Kong, 2019; Purbrick, 2019).

According to the Digital 2022 survey findings, 88% of Hong Kong residents utilize online social media, mostly to interact with friends and family members (Kemp, 2022). Compared to other age groups, young people are more media-savvy and are thus more likely to use new information and communication technologies (Palfrey and Gasser, 2013). This behavior has given rise to social media addiction that, according to Shek and Yu (2016), affects 17–26% of teenagers in Hong Kong. Moreover, available evidence points to positive associations between social media use and radical views (Lee, 2018), radical participation (Zhu et al., 2020), network activism (Ting, 2019), and street politics (Wang, 2018). Prior studies on social media use examined primarily on actual news consuming *via* social media, which is composed of both active and passive news consumption. However, the unique effect of intention to consume news *via* personal social media network on political trust of Hong Kong youth remains insufficiently explored.

## 3. Intention to consume news *via* personal social media network and political trust: Theories

A large research agenda on selective exposure and high-choice media effects in the 21st century has criticized the “echo chambers” and homogeneous news consumption assumption. Most of them suggest people actively engaging into politics (i.e., those with a strong intention to consume political news) are interested in not only like-minded information but also attitude-inconsistent views (Weeks et al., 2016; Bail et al., 2018; Peacock et al., 2021). However, the discussion of these research regards intention to consume consumption as a binary interaction between individual and social media sites without taking into account lifestyle and interpersonal factors (DellaPosta et al., 2015; Weeks et al., 2016; Bail et al., 2018; Guess, 2021; Peacock et al., 2021).

As Song et al. (2021) observed, one of the primary reasons for utilizing social media platform is to maintain relationships with peers and families, especially for the younger generation. Moreover, Song et al. (2021) further argued that passive forms of news consumption (i.e., “reliance on peer or informed news”) may increase the negative consequence of political cynicism, but active forms might not. Intention to consume news *via* personal social media network leads a series of activities such as persistent information endorsement, sharing, and discussion. The information obtained *via* personal social media network is more likely to exert influence on users’ views as social media sites are increasingly viewed as spaces for personal identity construction (Mummolo, 2016). Active social media users who consider themselves to be highly influential opinion leaders are more likely to try to influence others to change their political attitudes and behaviors both directly and indirectly (Weeks et al., 2015). To sustain this personal influence and extend its reach within the online community, social media users are unlikely to ignore attitude-inconsistent information once that information has been followed, commented on, liked or disliked by their friends. In particular, they will assign greater value to such information if it is endorsed by a friend who has social influence and is seen as an opinion leader in their network. In other words, the importance and relevance of a topic may outweigh the discomfort of discussing attitude-inconsistent issues. This assertion is confirmed by the findings reported by Anspach (2017), who assessed intention to consume news on Facebook, indicating that social media endorsement features can make political news more visible to millions of entertainment-seekers who might otherwise ignore it. Moreover, the author noted that when the information is endorsed by their family members and friends, rather than by unknown users, individuals are more likely to consume news *via* social media regardless of belief congruency (Anspach, 2017).

In sum, intention to consume news *via* a personal social media network can motivate users to take seriously and review critically counter-attitudinal information, which will reduce the likelihood of opinion polarization, and promote understanding of complex politics while building trust in the current governing system (Slater, 2007; Jamieson and Cappella, 2008). Thus, as a part of the present study, data collected *via* a three-wave survey among Hong Kong youth was analyzed to validate this hypothesis.

## 4. Important covariates of political trust

### 4.1. Traditional media

Information in the traditional media normally flows one-way, from the top level (government, editors, and professionals) to ordinary users at a lower level; therefore, users have few opportunities to offer their feedback and share their opinions with others (Ceron and Memoli, 2016). For a long time, government agencies have cooperated with traditional media to disseminate important announcements, promote public policies, and clarify standpoints favorable to the regime, in the belief that this strategy will increase public trust and confidence in political institutions and systems (Norris, 2000; Tewksbury and Rittenberg, 2012). Indeed, research on this topic indicates presence of a positive relationship between traditional media use and political trust in general (Strömbäck et al., 2016). However, in some cases, political information disseminated *via* TV channels can lead to a low level of political trust (Norris, 2000; Avery, 2009; Aarts et al., 2012), especially during elections when focus is given to political debates (Cappella and Kathleen, 1997; Avery, 2009).

### 4.2. New media on the internet

The aforementioned relationship between intention to consume news *via* traditional media and political trust was disrupted by the emergence of new information and mobile technologies that have diversified the media landscape, such as blogs, streaming, e-journals, and social media sites (e.g., Facebook, Twitter), which provide users greater access to news and information at a far lower cost. These widespread new medias have revolutionized the “representative journalism” (Hartley, 2000) by enabling interpersonal interactions (Chao-Chen, 2013). While this development is largely welcomed, some scholars argue that unauthorized media platforms undermine the political advocacy function played by the traditional media (Benkler, 2006; Bakshy et al., 2012) that promoted a multi-polar information environment, and thus may undermine political trust (Zhang and Guo, 2021). To counteract this impact, traditional mainstream and political accounts have also become active in extending their influence to the online community by running mobile applications and official channels on new medias (Halpern and Gibbs, 2013; Enli, 2017). Consequently, there is a growing consensus that the information shared online may still be controlled by the political elite and mediated by editors, thereby influencing political understanding, satisfaction, and trust in the current regime (Tolbert and Mossberger, 2006; Ananny, 2014).

### 4.3. Political interest

Findings reported in pertinent literature suggest that political interest is positively associated with both the intention to consume news and political trust (Prior, 2007; Strömbäck, 2015; Claes and Hooghe, 2017). However, several authors have provided evidence indicating that political trust is primarily influenced by political interest (Aarts et al., 2012; Albæk et al., 2014). Furthermore, Strömbäck et al. (2016) purported that, even after controlling for political interest, the intention to consume news still has a noticeable effect on political trust.

Since all three aforementioned factors (intention to consume news *via* traditional media, intention to consume news *via* new media on the Internet, and political interest) are documented to be associated with political trust, in the present study, they are included as control variables in order to explore the unique effect of intention to consume news *via* personal social media network and political trust.

## 5. Materials and methods

### 5.1. Procedures

Following previous studies on media and politics in which young people were defined as those aged 16–24 years, we targeted the same age group in Hong Kong for our data collection (Bakker and De Vreese, 2011; Lee and Chan, 2012). To ensure that the study sample was representative of this subpopulation of Hong Kong, we adopted a two-stage stratified sample design, as this is a sampling method frequently used by the Census and Statistics Department of Hong Kong. In the first stage, we selected a random sample of households based on geographical area and neighborhood type. In the second stage, from each household, one member aged 16–24 (as determined by their last birthday), if available, was invited to partake in the survey.

For this purpose, we prepared a paper-based questionnaire featuring a clearly stated research purpose on the first page. Only young people who gave their formal informed consent (which was sought from the parents for those under 18 years of age) proceed with the survey, which they completed in the presence of researchers, who were available to answer any questions. Three waves of data collection were conducted, coinciding with large-scale social movements in which many young people actively participated. Thus, the first survey wave was conducted in October 2015, 10 months after the Umbrella Movement had ended; the second wave was conducted in October 2016, 8 months after the serious Mong Kok violent conflicts; and the third wave was completed in April 2017, when the Hong Kong Chief Executive election had been completed and the voters had not yet achieved universal suffrage.

The first wave of data collection included 616 young people (56.9% of whom were male). However, 216 and further 184 failed to complete the second and third wave, respectively, resulting in only 216 complete individual datasets available for analysis. The characteristics of the final sample that participated in all three waves are provided in Table 1. Although attrition was significant, further analyses did not uncover significant differences between the remaining participants and the dropouts.

**Table 1:** Descriptive statistics for participants in three waves (*N* = 216).

	Wave 1	Wave 2	Wave 3
	Mean (Standard deviation)/Percentage		
Age	18.81 (2.70)	19.81 (2.70)	20.03 (2.79)
Range of age	16–24	17–25	17–26
Male	56.9%	56.9%	56.9%
Education level			
Secondary school	75.0%	51.4%	50.5%
Postsecondary school	18.1%	37.5%	42.6%
Not provide the answer	6.9%	11.1%	6.9%
Economic status			
Employee	22.7%	29.2%	23.6%
Self-employed	0.5%	0.0%	0.0%
Student	71.8%	67.6%	74.1%
Unemployed	1.9%	2.3%	2.3%
Not provide the answer	3.2%	0.9%	0.0%

### 5.2. Measures

A single item was used to assess the intention to consume news from a personal social media network (de Zúñiga et al., 2014; Ekström and Östman, 2015). Young people were asked, “On a typical day, how often do you use social media sites such as Facebook, LINE, and WhatsApp to get news about current political events from your friends and family members?” The options were scaled from 1 (never) to 5 (very often).

For measuring political trust, following the strategy adopted by Ceron (2015), we employed three items, pertaining, respectively, to the respondents’ degree of trust in Hong Kong’s political parties, the Hong Kong Legislative Council, and the Hong Kong government. Their responses were given on a five-point scale, ranging from 1 (no trust at all) to 5 (complete trust).

For obtaining data pertaining to covariates, we adopted three items from the survey conducted by Dimitrova et al. (2014), pertaining to the intention to consumer news *via* the newspapers, TV, radio, and the internet. For each item, participants were instructed to select one of the 11 options (15 min or less, 16–30 min, 31 min to 1 h, 61 min to 1.5 h, 91 min to 2 h, 121 min to 2.5 h, 151 min to 3 h, 181 min to 3.5 h, 211 min to 4 h, 241 min to 4.5 h, and more than 4.5 h) to report how much time they spent reading newspaper news, listening to radio news, watching television news, and reading, listening, and watching online news in a typical day. These options were coded from 1 to 11.

Finally, three questions were used to assess the participants’ political interest based on the measurements developed by Boulianne (2011), namely (1) “Are you interested in information about what is going on in government and politics?” whereby options were scaled from 1 (not at all interested), to 5 (extremely interested); (2) “How closely do you pay attention to information about what is going on in government and politics?” with the responses ranging from 1 (not closely at all) to 5 (extremely closely); and (3) “How often do you pay attention to what is going on in government and politics?” where options were scaled from 1 (never) to 5 (all the time).

### 5.3. Data analysis

To address the measurement issues, we performed confirmatory factor analysis (CFA) on the data obtained in each wave to establish three latent variables. Specifically, we tested if the intention to consume news *via* traditional media can be loaded on the intention to consume news through newspapers, radio, and TV; we evaluated if political trust can be loaded on trust in Hong Kong political parties, the Hong Kong Legislative Council, and the Hong Kong Government; and we assessed if political interest can be loaded on the three relevant items. Due to the small sample size, we did not integrate the measurement model into the main analysis. Instead, factor scores for the three waves were computed and saved.

In the main analysis, we tested four hypotheses by fitting a cross-lagged model to the survey data. In the hypothesized model, we drew links from political interest to the intention to consume news *via* three channels (i.e., traditional media, new media on the Internet, and personal social media network), as well as those linking the intention to consume news *via* three channels to political trust. We also drew a direct link from political interest to political trust, and a series of links from the prior status of a variable to its later status. The Amos 26 (IBM New York) was adopted to assess the hypothesized cross-lagged model. To ensure that the model remains theory-driven, we were careful to add links based on the modification indices. The unstandardized pathway from Wave 1 to Wave 2 was constrained to be equal to that from Wave 2 and Wave 3 for all links.

## 6. Results

The CFA results indicate that the hypothesized factorial structure was well supported by the data gathered in the three survey waves [Wave 1: Chi-squared (df = 25) = 52.191; GFI = 0.949; RMSEA = 0.071; Wave 2: Chi-squared (df = 26) = 40.875; GFI = 0.959; RMSEA = 0.052; Wave 3: Chi-squared (df = 26) = 67.986; GFI = 0.936; RMSEA = 0.087]. The factor loadings for the measurement models at the three waves are reported in Table 2. All factor loadings were highly significant and larger than 0.40.

**Table 2:** Factor loadings in the measurement models (*N* = 216).

Items	Wave 1	Wave 2	Wave 3
News consumption from the traditional media			
Newspaper	0.82	0.64	0.69
Radio	0.54	0.76	0.74
TV	0.58	0.42	0.77
Political trust			
Trust in Hong Kong political parties	0.90	0.78	0.93
Trust in Hong Kong Legislative Council	0.78	0.85	0.81
Trust in Hong Kong Government	0.69	0.67	0.76
Political interest			
Are you interested in information about what is going on in government and politics?	0.86	0.78	0.92
How closely do you pay attention to information about what is going on in government and politics?	0.92	0.90	0.87
How often do you pay attention to what is going on in government and politics?	0.83	0.77	0.92

All factor loadings are highly significant (*p* < 0.001).

**Figure 1:**
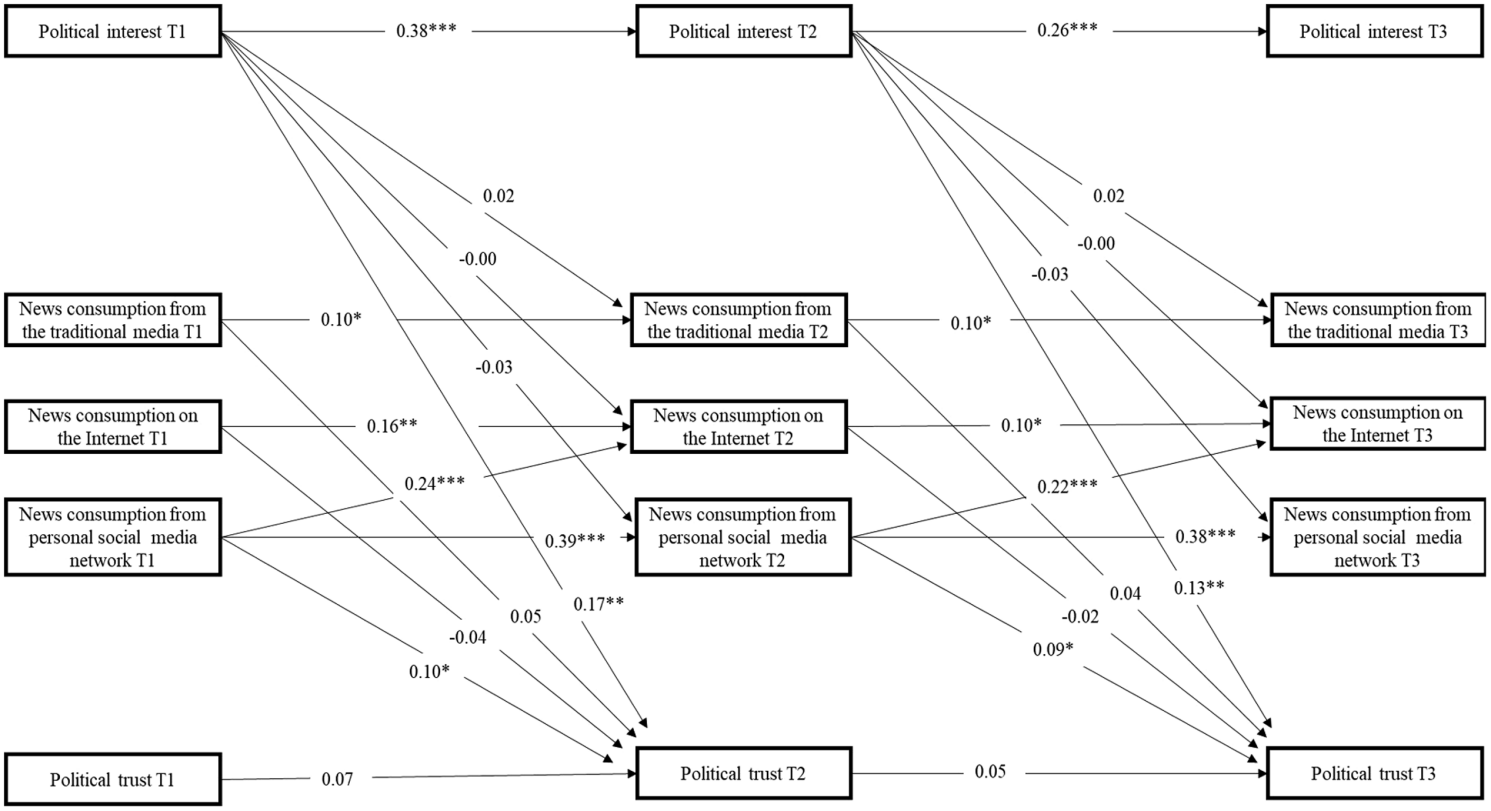
Results of the model assessing the intention to consume news *via* personal social media network on political trust (*N* = 216). *p < 0.05, **p < 0.01, ***p < 0.001. T1 = Time 1; T2 = Time 2; T3 = Time 3.

The computed factor scores were used to evaluate the cross-lagged models. The finalized model results are reported in Figure 1 and show acceptable model−data fit [Chi-squared (df = 80) = 236.562; GFI = 0.88; RMSEA = 0.095]. After controlling for prior status of political trust and political interest, the intention to consume news *via* personal social media network was the only variable with a positive and significant effect on political trust (*β* = 0.10, *p* < 0.05; *β* = 0.09, *p* < 0.05). In comparison, the intention to consume news from the internet and the traditional media did not have any significant effect on political trust. Political interest was found to significantly and positively influence political trust in a direct way (*β* = 0.17, *p* < 0.01; *β* = 0.13, *p* < 0.01). Moreover, our data analysis revealed that intention to consume news *via* personal social media network could facilitate the intention to consume news on the internet (*β* = 0.24, *p* < 0.001; *β* = 0.22, *p* < 0.001).

Political trust at T1 was found to insignificantly predict that at T2 (*β* = 0.07, *p* > 0.05). Political trust at T2 was found to insignificantly influence that at T3 (*β* = 0.05, *p* > 0.05). The findings are as expected because the data were collected in a time period during which a number of political events took place and political trust of young people experienced a tremendous fluctuation. The unstable political trust across three waves was actually an additional advantage of exploring the relative importance of different factors (e.g., intention to consume news *via* channels and political interest) in influencing the political trust of young people.

## 7. Discussion

As a part of the present study, we analyzed a three-wave dataset collected from a representative sample of Hong Kong’s youth to assess the role played by the intention to consume news *via* personal social networks in predicting political trust. Our findings indicate that news intentionally obtained from friends and family members within a personal social media network improved political trust, suggesting a relatively weak media effect and a relatively strong selection effect.

The results show that, in the current high-choice media environment, as users have diverse means of accessing news, the effect of each source of information may be weakened (Strömbäck et al., 2016). Thus, it is increasingly challenging for the political elite to establish influential power among young people and build their political trust. However, when young people utilize the internet for political purposes and intentionally consume news, there is no need for policy makers to be concerned about the influence of anti-government information affecting political trust, as the traditional media and government channels on the internet and the other new online media sources seem to be unable to effectively shape political trust. Although Government sectors and traditional mainstream news corporations are active in running official channels on a range of new media sites, the results of our model clearly show that they possess little capacity to affect political trust (Ananny, 2014; Zhang and Guo, 2021).

Motivated by reporting of Anspach (2017) that the intention to consume news through personal social media networks promotes perusal of different opinions, resulting in a dampened partisan orientation, our study appears to be the first to use the panel data to link news intentionally obtained from friends and family through social media to political trust. Akin to the heterogeneous information to which users of the new media (e.g., blog, Line TV) are exposed through the internet, information emerging from their personal social media networks is also diversified. However, in this context, social media users may choose not to ignore information which does not align with their views. The individual’s intention to consume attitude-inconsistent information from friends may be regarded as an opportunity to better understand their peers, which is a crucial step in establishing personal influence in the online network (Weeks et al., 2015; Mummolo, 2016). Thus, a non-radical and centralized belief system and trust in the political system may still be maintained after carefully reviewing all news endorsed by peers, no matter whether the information is “liked” or “disliked.” Our data collection waves were deliberately chosen to coincide with important social movements in Hong Kong, which prompted discord among families and friends due to their different political orientations. Our findings suggest that this should not be a concern, as such conflicts are much better than ignorance in terms of building trust in politics within the broader society, introducing an interesting avenue for policymakers and educators to build political trust among young people.

Indeed, previous research reveals that “actual news consumption” (mostly referring to passive news consumption) *via* social media is usually linked to opinion polarization and reducing political trust among young people in Hong Kong (Jost et al., 2018; Zhu et al., 2019, 2020; Boulianne et al., 2020; Zhu and Chou, 2022). Nevertheless, this research suggests that “the intention to consume news” *via* personal social media network is able to slightly promote young people’s political trust. Therefore, it is interesting that policy makers can opt for either increase censorship of social media to avoid political polarization (inspired by the effect of passive news consumption on extreme political attitudes in the literature) or use social media as an education platform to promote political trust (inspired by this study). This can be a quite valuable research question for future studies to further explore.

Overall, our results highlight the importance of intention to use personal social media network to get news in building political trust. They also suggest that there is no need to fear information that is unfavorable to the regime being disseminated *via* the internet, as the new medias are open to all sources and are strong enough to balance the impact of negative voices.

## 8. Limitations

Although this study contributes to both theoretical and policy implications, its limitations must be considered when interpreting the reported findings. In particular, due to high attrition, the final sample is quite small for precluding integration of measurement models into the structural model and perform full structural equation modeling. In addition, only one subjective item was adopted to measure respondents’ intention to consume news from the internet and their personal social media network, which made it inadequate to analyze and discuss measurement issues further (Guess, 2015). Thus, these limitations should be addressed when replicating or extending this study to other societies. Future investigation on the link between the intention to consume news and political trust could consider employing experience sampling and objective observation through tracking software as options.

## Data availability statement

The raw data supporting the conclusions of this article will be made available by the authors, without undue reservation.

## Ethics statement

Ethical approval was obtained from The Education University of Hong Kong. Informed consent was obtained from all individual participants included in the study. Written informed consent to participate in this study was provided by the participants’ legal guardian/next of kin.

## Author contributions

YZ, ZT, and AZ contributed to conception and design of the study and wrote the first draft of the manuscript. YZ, JH, and AZ contributed to the methodology section. YZ, ZT, and ZZ performed the statistical analysis. JH, YZ, ZZ, and ZT reviewed and edited the manuscript. YZ contributed to the funding acquisition of this study. All authors contributed to the article and approved the submitted version.

## Funding

This study was funded by grants from the Public Policy Research Funding Scheme, Hong Kong SAR (Project Number 2014.A5.006.15A) and the Hong Kong Scholars Programme, Society of Hong Kong Scholars and China National Postdoctoral Council.

## Conflict of interest

The authors declare that the research was conducted in the absence of any commercial or financial relationships that could be construed as a potential conflict of interest.

## Publisher’s note

All claims expressed in this article are solely those of the authors and do not necessarily represent those of their affiliated organizations, or those of the publisher, the editors and the reviewers. Any product that may be evaluated in this article, or claim that may be made by its manufacturer, is not guaranteed or endorsed by the publisher.
